# The accuracy and stability of the maxillary position after orthognathic surgery using a novel computer-aided surgical simulation system

**DOI:** 10.1186/s12903-019-0711-y

**Published:** 2019-01-15

**Authors:** Ju-Won Kim, Jong-Cheol Kim, Chun-Gi Jeong, Kyeong-Jun Cheon, Seoung-Won Cho, In-Young Park, Byoung-Eun Yang

**Affiliations:** 1 Division of Oral and Maxillofacial Surgery, Hallym University Sacred Heart Hospital, 11, Gwanpyeong-ro 170beon-gil, Dongan-gu, Anyang-si, Gyeonggi-do, 14066 Anyang, Republic of Korea; 2 Mir Dental Hospital, 12 Gongpyoungro Jung-gu Daegu, 41940 Daegu, Republic of Korea; 3 Division of Orthodontics, Hallym University Sacred Heart Hospital, Anyang, Republic of Korea; 4 Graduate School of Clinical Dentistry, Hallym University, Chuncheon, Republic of Korea; 5 Institute of Clinical Dentistry, Hallym University, Chuncheon, Republic of Korea

**Keywords:** Orthognathic surgery, Virtual surgery, Computer-aided surgical simulation, Patient-customized osteotomy guide (PCG), Patient-customized Miniplate (PCM)

## Abstract

**Background:**

Many reports have been published on orthognathic surgery (OGS) using computer-aided surgical simulation (CASS). The purpose of this study was to evaluate the accuracy of the maxillary repositioning and the stability of the maxilla in patients who underwent OGS using a newly developed CASS program, a customized osteotomy guide, and a customized miniplate.

**Methods:**

Thirteen patients who underwent OGS from 2015 to 2017 were included. All patients underwent a bimaxillary operation. First, a skull-dentition hybrid 3D image was rendered by merging the cone beam computed tomography (CBCT) images with the dentition scan file. After virtual surgery (VS) using the FaceGide® program, patient-customized osteotomy guides and miniplates were then fabricated and used in the actual operation. To compare the VS with the actual surgery and postoperative skeletal changes, each reference point marked on the image was compared before the operation (T0) and three days (T1), four months (T2), and a year (T3) after the operation, and with the VS (Tv). The differences between ΔTv (Tv-T0) and ΔT1 (T1-T0) were statistically compared using tooth-based reference points. The superimposed images of Tv and T1 were also investigated at eight bone-based reference points. The differences between the reference points of the bone surface were examined to evaluate the stability of the miniplate on the maxilla over time.

**Results:**

None of the patients experienced complications. There were no significant differences between the reference points based on the cusp tip between ΔTv and ΔT1 (*p* > 0.01). Additionally, there were no significant differences between the Tv and T1 values of the bone surface (p > 0.01). The mean difference in the bone surface between Tv and T1 was 1.01 ± 0.3 mm. Regarding the stability of the miniplate, there were no significant differences between the groups. The difference in the bone surface between T1 and T3 was − 0.37 ± 0.29 mm.

**Conclusions:**

VS was performed using the FaceGide® program, and customized materials produced based on the VS were applied in actual OGS. The maxilla was repositioned in almost the same manner as in the VSP plan, and the maxillary position remained stable for a year.

## Background

Skeletal malocclusion affects oral health and is highly associated with dental trauma and masticatory difficulties as secondary effects of parafunction and teeth crowding [1]. Orthognathic surgery (OGS) is used to resolve imbalances involved in the craniofacial structure and skeletal malocclusion, thereby improving the oral and facial function and aesthetics of the patient. The efforts of both orthodontists and surgeons can dramatically improve the quality of life of patients experiencing functional and aesthetic discomfort. However, jaw misplacement by a surgeon during OGS is difficult for an orthodontist to revise after the operation. During traditional bimaxillary OGS, the maxilla is first moved, and the mandible is relocated relative to the maxilla. Therefore, it is most important to move the maxilla to a planned position during OGS. Efforts to achieve such outcomes include freehand relocation [2] and the use of an internal reference point, which are currently applied by several surgeons. However, external reference points are the most accurate method to use during LeFort I osteotomy [3]. In recent years, the progress in OGS has mainly resulted from the use of a virtual surgery plan (VSP) to accurately reposition the bone segments [4]. There are problems associated with conventional OGS and several reasons why VSPs are favored. An analysis of dentofacial deformity is based on the information obtained through several preoperative examinations. Once the analysis is completed, subsequent surgical planning is initiated using a visual treatment objective (VTO), which determines where each component should be positioned in relation to the fixed reference structure (skull base) and another. When the VTO involves the movement of only a single jaw, either the maxilla or mandible, a simple hinge articulator is sufficient for mock surgery. However, when the VTO involves the movement of both jaws, a semi-adjustable articulator is used as these articulators can better reproduce the centric relation (CR) and centric occlusion within an acceptable anatomical average. The most difficult aspect in performing model surgery is in the repositioning of the maxillary cast during bimaxillary surgery [5]. After the mock surgery is performed according to the surgical plan, two surgical occlusal splints (an intermediate splint (IMS) and a final splint) are made for bimaxillary surgery. Occlusal splints (or wafers) locate the dental arches in any preplanned relationships and eliminate unreliable intraoperative guesswork [6]. As the first step in simulating a bimaxillary surgery, a face-bow transfer procedure is required to transfer the maxilla to a semi-adjustable articulator. However, it is impossible to transfer the patient’s maxillary dentition to the articulator and accurately reproduce the patient’s anatomy [7–10]. In addition, it is difficult to achieve complete three-dimensional (3D) movement in a model surgery in cases of patients with severe facial anomalies, even though the face-bow is used to correctly reproduce the patient’s actual maxillary position in the articulator. During the model surgery, the upper arm of the semi-adjustable articular is used as a reference for moving the maxillary cast. However, the most common technique for repositioning the maxilla in the operating room is the use of an external reference point with the help of the IMS. Therefore, the substantive reference for repositioning the maxilla is the mandible. In most cases, OGS requiring maxillary movement is performed under general anesthesia. Some researchers have reported that the position of the mandible deviates from its normal position under general anesthesia [11, 12]. Even if a face-bow transfer is performed well enough to accurately reflect the anatomy of the patient, the model surgery is performed well, and the IMS is made perfectly, errors may occur when the surgeon uses the mandible as a reference and repositions the maxilla using only the IMS. Therefore, considerable time is required to determine the desired position of the maxilla when conventional bimaxillary surgery is planned. Recently, computer-aided surgical simulation (CASS) and device manufacturing using computer-aided design (CAD) and computer-aided manufacturing (CAM) technologies have attracted attention for precise OGS [13, 14]. Herein, we performed a VS using FaceGide® (MegaGen Co., Daegu, Korea), a CASS program, instead of a model surgery and face-bow transfer in preparation for OGS. In addition, patient-customized miniplates (PCMs) were used instead of the IMS. The purpose of this study was to evaluate the surgical accuracy and long-term stability of maxillary repositioning using the FaceGide® system by comparing cone beam computed tomography (CBCT) images over time.

## Methods

### Subjects

Thirteen patients were selected from a list of medical records. The sample consisted of seven females and six males with a mean age of 22.9 ± 3.3 years (range, 18–29). The patients were selected according to the following inclusion criteria: (1) patients who underwent surgery between February 2015 and August 2017; (2) those who completed presurgical orthodontic treatment; and (3) patients who underwent bimaxillary surgery to treat skeletal malocclusion. The exclusion criteria were as follows: (1) patients with cleft palate or craniofacial anomalies; (2) patients who had not undergone surgery with FaceGide®; and (3) patients who were unwilling to participate in this study. All medical practices conformed to the Declaration of Helsinki as a medical protocol. The study protocol was approved by the hospital’s Institutional Review Board (IRB No. 2018–06-016). All patient data were anonymized and de-identified prior to the analysis. Detailed patient characteristics are presented in Table 1.

**Table 1:** Patient characteristics and surgery descriptions

Patient No.	Age	Sex	Characteristics	Surgery
Pt 01	28	F	FA (Lt side^a^), Angle III	Lefort I, BSSO, Genio
Pt 02	24	F	FA (Rt side^a^), Angle I	Lefort I, BSSO
Pt 03	24	M	Angle III	Lefort I, BSSO, Genio
Pt 04	29	M	FA (Lt side^a^), Angle III	Lefort I, BSSO
Pt 05	24	F	FA (Rt side^a^), Angle I	Lefort I, SSO(Rt), HRO(Lt), Genio
Pt 06	20	M	FA (Lt side^a^), Angle III	Lefort I, HRO(Rt), SSO(Lt), Genio
Pt 07	20	M	FA (Lt side^a^), Angle III	Lefort I, BVSRO
Pt 08	26	F	Angle III	Lefort I, BSSO
Pt 09	22	F	FA (Rt side^a^), Angle III	Lefort I, BSSO
Pt 10	18	F	FA (Lt side^a^), Angle III	Lefort I, BSSO
Pt 11	21	M	FA (Rt side^a^), Angle III	Lefort I, BSSO
Pt 12	21	M	FA (Rt side^a^), Angle III	Lefort I, BSSO
Pt 13	21	F	FA (Rt side^a^), Angle I	Lefort I, BSSO

*FA* Facial asymmetry, *Angle* Angle malocclusion classification, *Lefort I*, Lefort I osteotomy, *BSSO* Bilateral sagittal split ramus osteotomy, *HRO* Horizontal ramus osteotomy, *Genio* Genioplasty, *BVSRO* Bilateral verticosagittal ramus osteotomy. ^a^The direction indicated in parentheses following FA is the deviation direction

### Preoperative procedures and virtual OGS

**Fig. 1:**
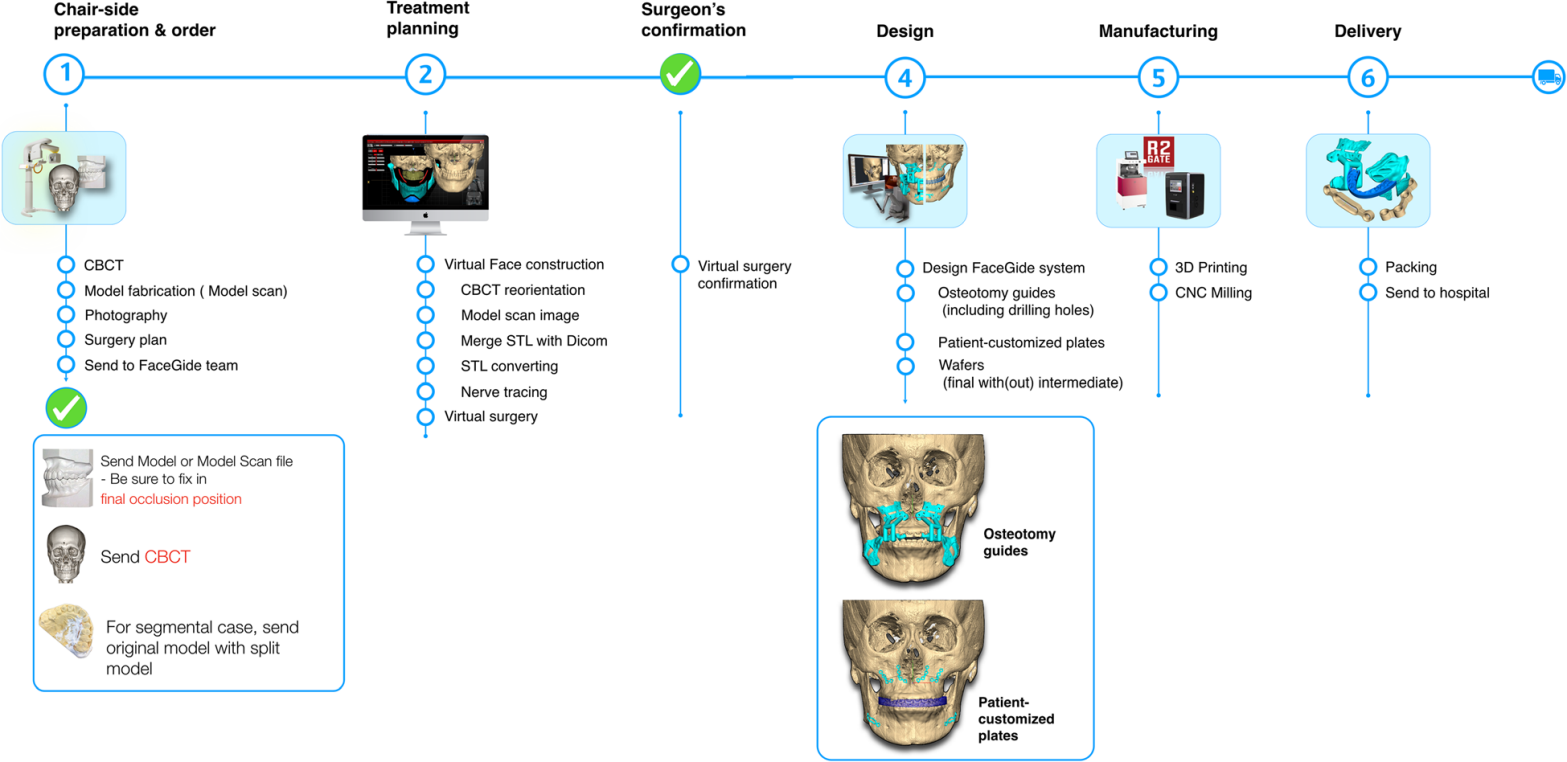
Workflow of orthognathic surgery using the FaceGide® system

**Fig. 2:**
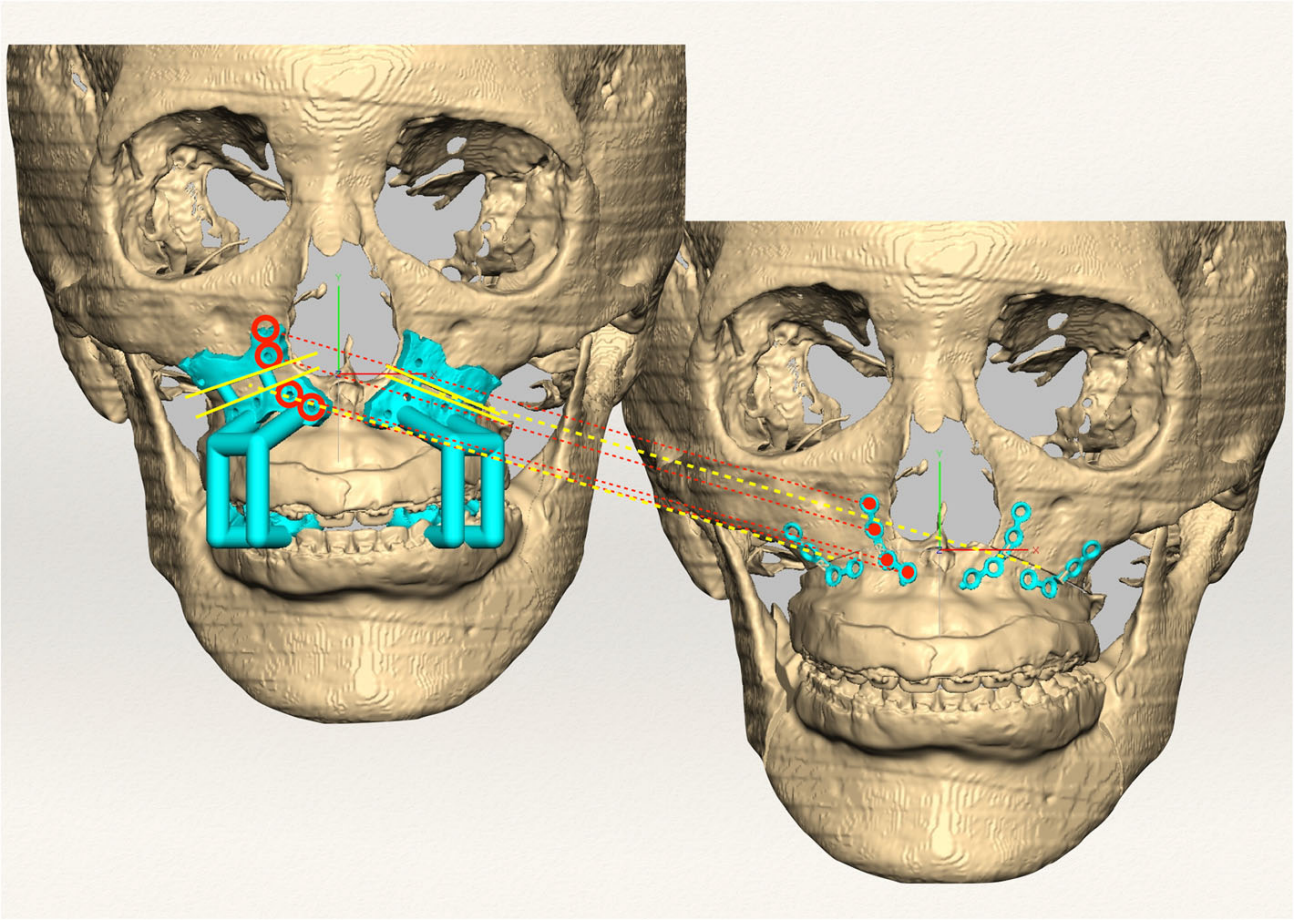
Patient-customized osteotomy guides (PCGs) and patient-customized miniplates (PCMs) designed for surgery (Pt 12 of Table 1). The patient had a longer maxilla on the right side; therefore, the amount of bone removed after the osteotomy was greater on the right side. The yellow lines indicate the osteotomy line. There were 16 holes in the PCGs for the insertion of the bone screws. The empty red circle is the location of the screw holes in the PCGs, and the red circle on the right is the site of screw insertion. In the virtual face on the right side (where the maxillary bone is moved), the screws are marked, and the PCGs were designed after the image was constructed based on the virtual face on the left side (without moving the maxilla)

Our protocol for OGS with the FaceGide® system was as follows (Fig. 1). Clinical photographs of the patient were taken after a clinical examination. CBCT (Alphard 3030, Asahi, Inc., Kyoto, Japan) was performed two weeks before the surgery to obtain a 3D image of the patient. All images were obtained with the Frankfort plane parallel to the horizontal plane, a field of view of 200 × 200 mm, a voxel size of 0.39 mm and exposure conditions of 80 kVp, 5 mA, and 17 s. The patients were scanned wearing a CR wax bite to ensure that their condyles were scanned in the CR position, that is, with the condyle resting in the glenoid fossa. With CBCT, a patient’s dental structure cannot be obtained accurately due to bracketing, blurring, and enlargement of the image. Almost all orthodontic patients wear fixed metal orthodontic appliances or have metallic brackets, which produce striped artifacts that distort the view of the dentition and occlusions during scanning. Therefore, a conventional impression was taken, and a pair of stone casts was fabricated for each patient. The surface image of the casts was then digitized into surface tessellation language (STL) format using a desktop model scanner (Freedom HD; Dof, Inc., Seoul, Korea). The CBCT images were transformed into DICOM format, and three-dimensionally reconstructed. Then, the CBCT reconstruction and the dental cast scan files were sent to the digital center. Subsequently, the DICOM and STL files were imported into a planning software program. The patient’s CBCT scan and the scanned image of the patient’s dental cast were registered. Semiautomatic merging started with registration of the image of the teeth obtained from the dental cast to the CBCT image of the teeth, which is relatively accurate. The images were merged process via manual registration by selecting three anatomical landmarks from the dentition. The contour of the dental cast image placed on the CBCT image was examined, and fine adjustments were made if necessary. The next step was reorientation of the skull image to reconcile the views of the surgeon, the orthodontist, and the digital technician. In this way, the final virtual hybrid skull-dentition 3D image (virtual face) was obtained. The reorientation image was sent to the surgeon, and telecommunication with the digital technician took place via the computer screen. The position of the osteotomy, the movement of the bone segment, the position of the customized plate, and the final occlusion were determined. After the final confirmations were made, virtual materials (such as the patient-customized osteotomy guides (PCGs), PCMs, and splints) were designed (Fig. 2). After the surgeon checked them, their images were exported as STL files. Then, the actual materials were produced by a rapid prototyping machine (S30 3D printer, Rapid Shape GmbH, Heimsheim, Germany) and a computer numerical control (CNC) machine (ARDEN, TPS Korea Ltd., Gwangju, Republic of Korea).

### Actual OGS

**Fig. 3:**
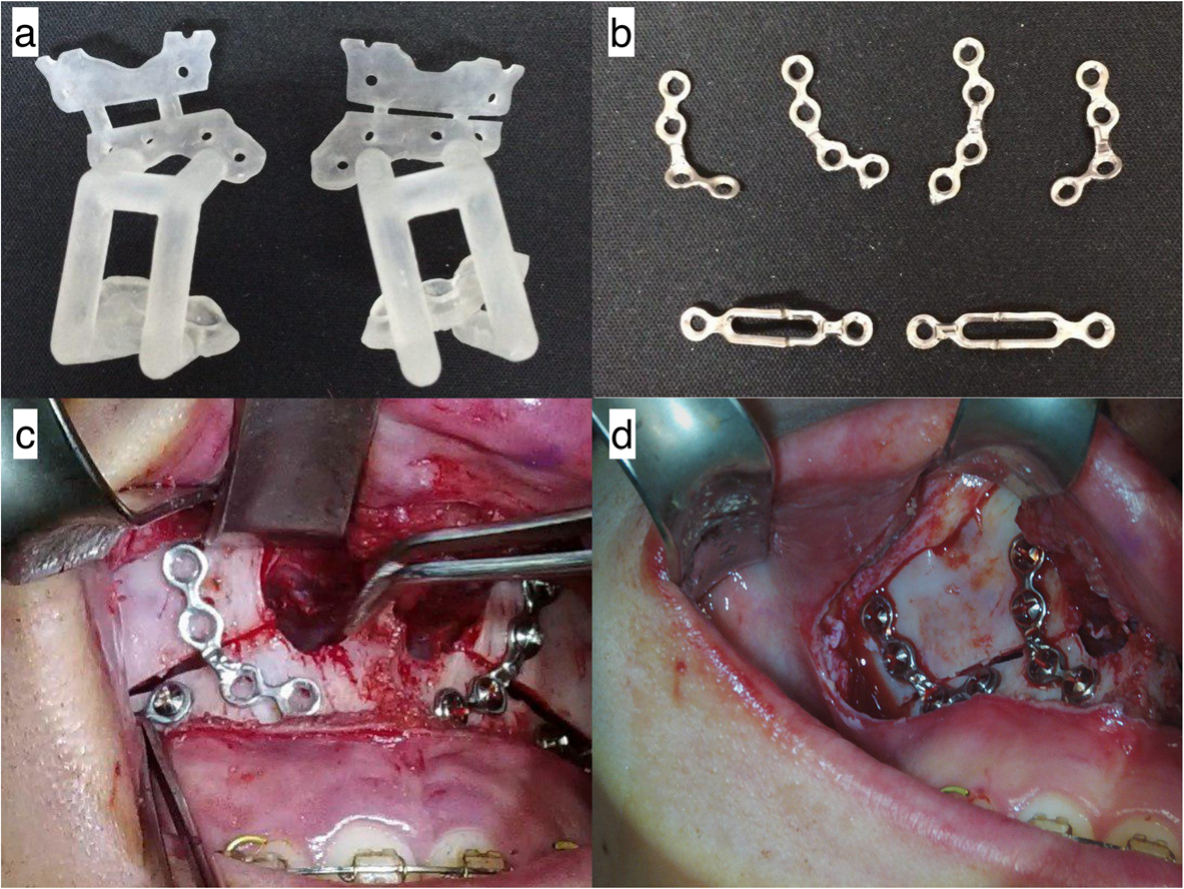
The actual maxillary osteotomy guides and patient-customized miniplates (PCMs) on the patient (Pt 12). (**a**) Osteotomy guides fabricated using a 3D printer. (**b**) PCMs. The PCMs below were used for the mandibular surgery. (**c**) The PCM was applied to the right maxilla. The holes in the PCMs were aligned with preformed holes on the bone surface. (**d**) PCMs in place

All physical components and reports regarding the VS showing the PCGs (including drilling holes) and PCMs were sent to the surgeon before the OGS. These materials were then delivered to the operating room for sterilization (Fig. 3a, b). The maxilla was repositioned and fixed with four L-shaped PCMs and monocortical self-drilling screws. The customized maxillary miniplates have holes corresponding to the drilling holes in the PCGs. As a result, bone holes are precisely formed using PCGs, and prefabricated miniplates are fitted to these holes (Fig. 3c, d). The PCM was positioned by merely inserting a self-drilling screw into the predrilled hole to ensure that there was no stress on the miniplate when the other screws were inserted. Mandibular surgery was performed in the same manner used for the maxilla, and customized mandibular miniplates corresponding to the osteotomy guides were used. After the ramus osteotomy, the distal segments of the mandible were repositioned using the final splint. Proximal segment positioning devices were used for proximal segment repositioning [15]. After maxillomandibular fixation (MMF) with rubber bands, the mandible was stabilized with customized miniplates. MMF was maintained for three days. Operations were performed by a single surgeon (BE.Y).

### Outcome evaluation

**Fig. 4:**
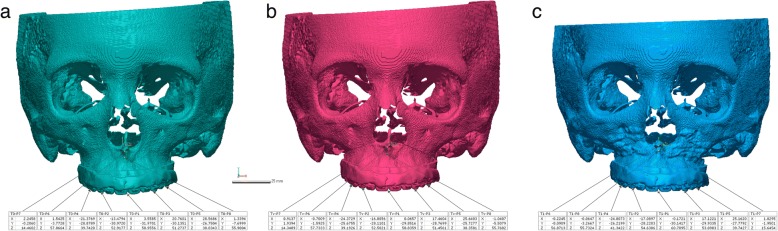
The coordinate value of each reference point at (**a**) T0, (**b**) Tv and (**c**) T1

**Fig. 5:**
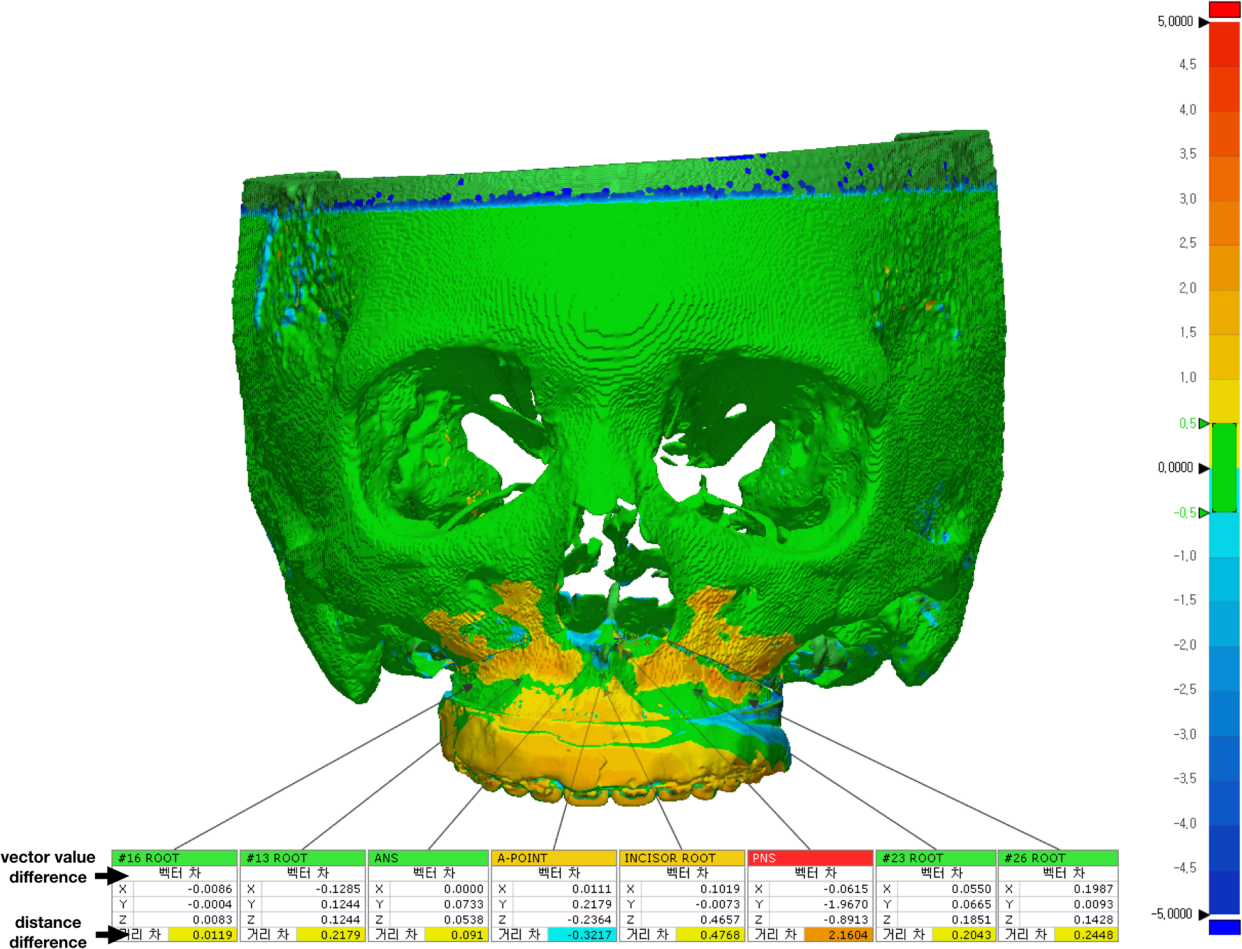
Comparison of reference points located on the bone surface after the superposition of Tv and T1 images

**Fig. 6:**
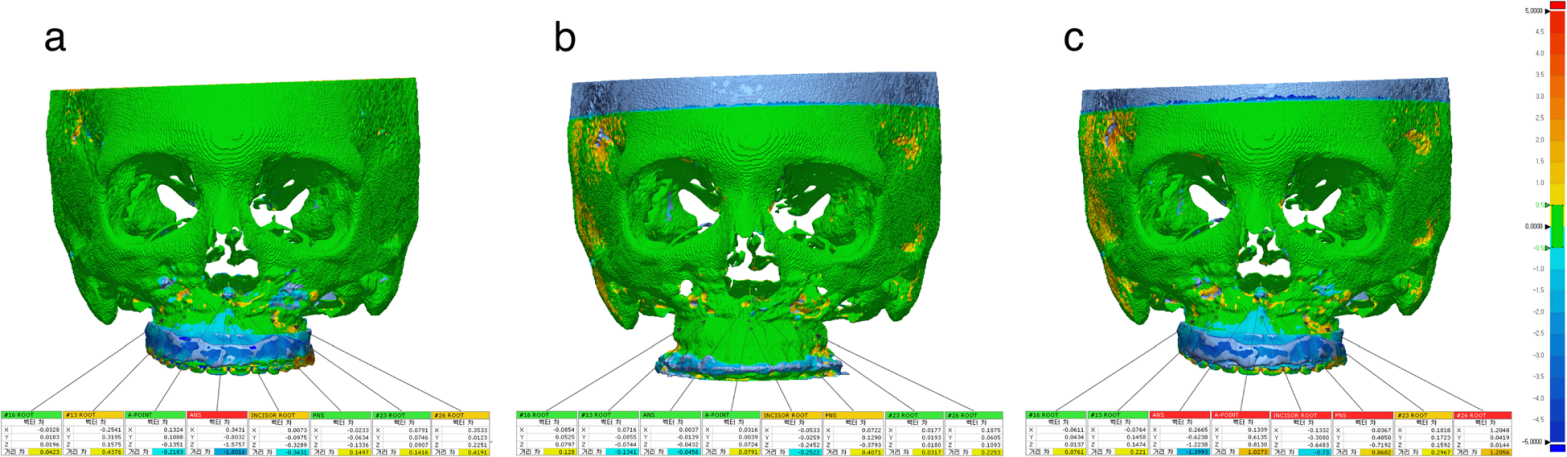
Comparison of reference points located on the bone surface after the superposition of (**a**) T1 and T2 images, (**b**) T2 and T3 images and (**c**) T3 and T1 images

3D CBCT images were obtained before the surgery (T0) and three days (T1), four months (T2) and one year (T3) after the surgery. The 3D image of the virtual OGS is denoted as Tv (T virtual). The predicted results and achieved outcomes were evaluated by comparing the eight landmarks (the center point between the cusp tips of the upper central incisors, the cusp tips of the upper canines, the mesiobuccal cusp tips of the upper first molars, the anterior nasal spine (ANS), the posterior nasal spine (PNS) and the A point) specified on the four sets of CBCT images. Each coordinate value was marked according to the trigonal system (x, y, z) and recorded in the program (Geomagic Freeform Plus, 3D Systems, North Carolina, USA) (Fig. 4). The X-axis represents the left and right directions, the Y-axis represents the up and down directions, and the Z-axis represents the front and back relationships. An individual t-test was performed for statistical comparisons between ΔTv (Tv-T0) and ΔT1 (T1-T0). The STL files from each period were evaluated using PolyWorks Inspector™ (InnovMetric Software, Inc., Quebec, Canada) to measure differences in the bone surface. Because superimposition must be performed based on a nonsurgically exposed region, such as the cranium, we used the virtually planned final position of the maxilla and the postoperative position of the maxilla for surface registration. Eight reference points were examined to compare the maxillary changes at T1, Tv, T2, and T3. T2 and T3 were times when orthodontic treatment had already begun. Therefore, measurements were based on the bone reference points rather than the tooth positions because the tooth positions changed at all time points. Measurements were made on the maxillary bone surface in front of the tooth root (the midpoint of the upper central incisors and canines and the mesiobuccal root of the first molars) instead of the cusp tip (5 sites) of the teeth among the eight reference points used in Geomagic Freeform Plus. The direct bone distance between the actual operation (T1) and VS (Tv) was measured (Fig. 5), and statistical comparisons between the coordinate values of the reference points were also investigated. Independent t-tests were used to compare values. Images were obtained at three time points (T1, T2, and T3) and then superimposed (Fig. 6). The three groups (T1 and T2, T2 and T3, T1 and T3) were compared to assess the postoperative stability of the maxilla. One-way ANOVA with Tukey’s multiple comparison tests was used for comparisons among the three groups. Data were analyzed using the Statistical Package for Social Sciences (SPSS, version 23.0, IBM Co., Armonk, NY, USA). *P*-values less than 0.01 were considered significant.

## Results

**Fig. 7:**
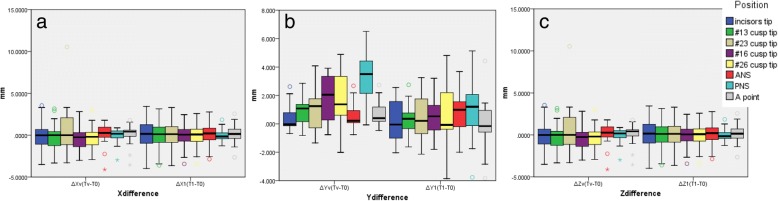
(**a**) Difference between ΔXv and ΔX1 on the X-axis. There was no significant difference between the two groups. (**b**) Difference between ΔYv and ΔY1 on the Y-axis. There was no significant difference between the two groups. (**c**) Difference between ΔZv and ΔZ1 on the Z-axis. There was no significant difference between the two groups

**Fig. 8:**
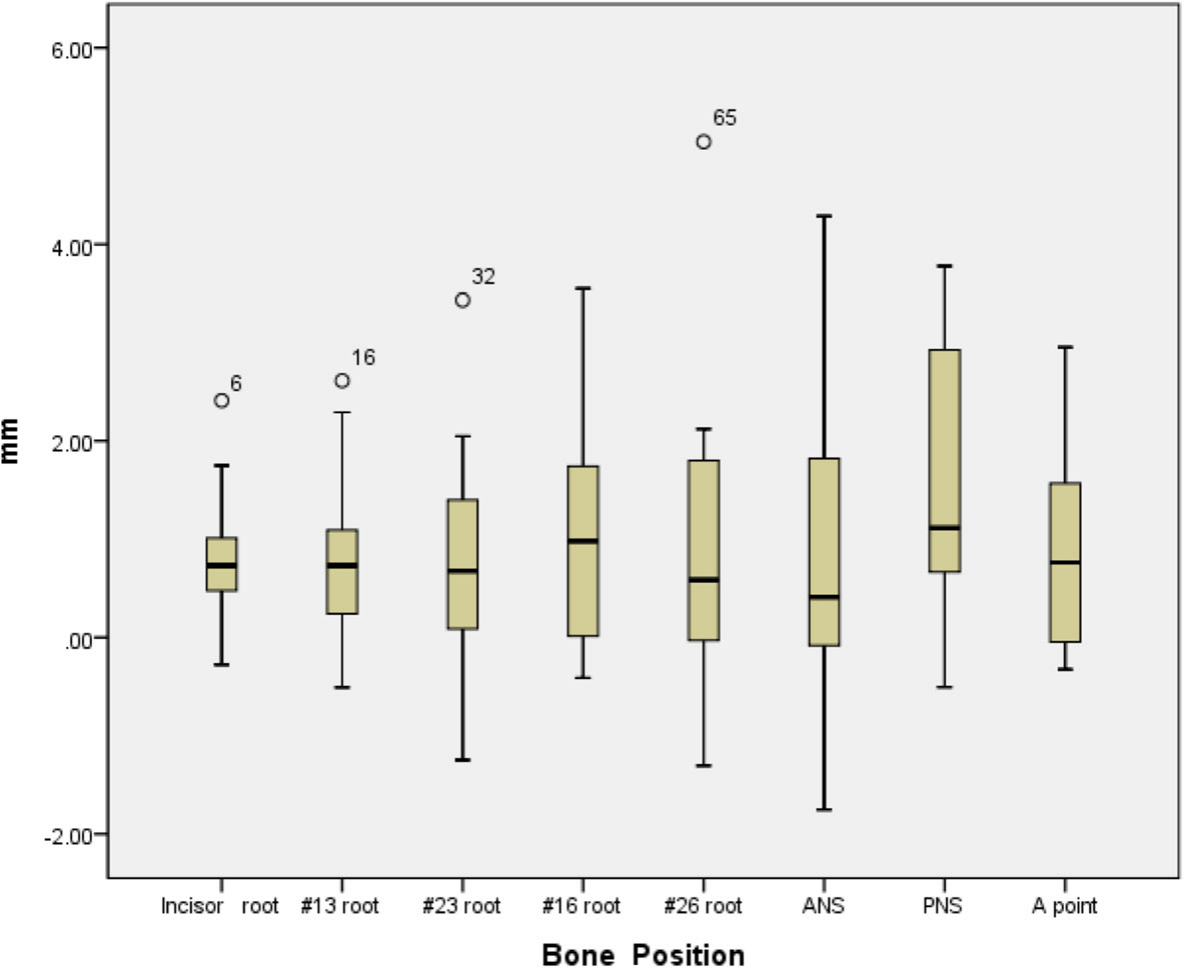
Differences between Tv and T1 at each bone reference point

**Fig. 9:**
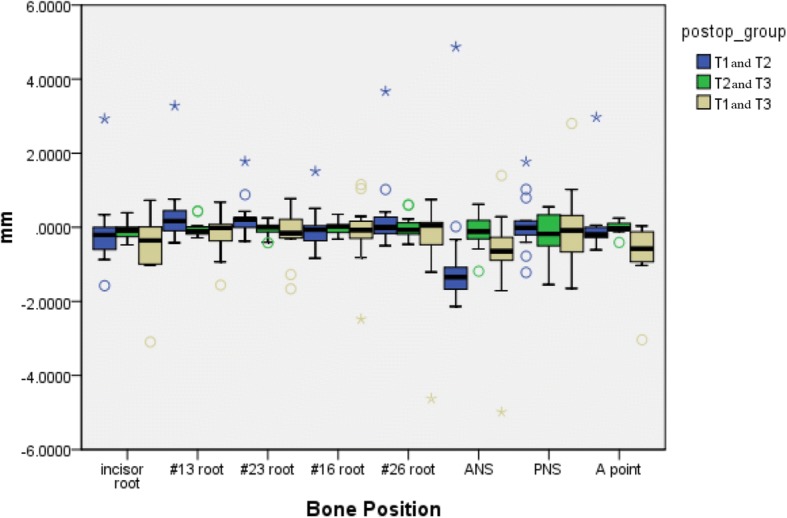
Comparison of the three groups after the superposition of T1, T2, and T3 images. There were no significant differences among the three groups

Satisfactory clinical outcomes were achieved in all patients, and VS was successfully reproduced in the actual surgery for all patients. All 13 patients were also satisfied by the postoperative results, including the occlusion and facial profile. No complications, such as malocclusion, tooth loss, sensory disturbance of the infraorbital nerve or infection, occurred during the follow-up period. The differences between ΔTv (Tv-T0) and ΔT1 (T1-T0) are shown in Table 2. There were no significant differences between the time points (Fig. 7). The Tv and T1 coordinate values for the bone reference points and the direct distances are shown in Table 3. There were no significant differences among the coordinate values. The mean distance difference at all reference points between Tv and T1 was 1.01 ± 0.3 mm (Fig. 8). The greatest difference was at the PNS, although the difference was not significant. There were no significant differences among the three groups (T1 and T2, T2 and T3, T1 and T3) regarding the stability of the maxilla. The mean difference between T1 and T3 was − 0.37 ± 0.29 mm (Table 4) (Fig. 9).

**Table 2:** Distance difference between ΔTv (Tv-T0) and ΔT1 (T1-T0)

		ΔXv (Tv-T0)		ΔX1 (T1-T0)			ΔYv (Tv-T0)		ΔY1 (T1-T0)			ΔZv (Tv-T0)		ΔZ1 (T1-T0)		
	n	Average	SD	Average	SD	*p*	Average	SD	Average	SD	*p*	Average	SD	Average	SD	*p*
Incisor tip	13	−0.004	2.097	0.003	2.293	0.994	0.468	1.075	0.216	1.426	0.616	0.034	0.370	1.166	1.649	0.024
#13 cusp tip	13	−0.059	1.909	− 0.071	2.075	0.988	0.910	1.042	0.271	1.226	0.165	0.148	0.522	1.334	1.515	0.014
#23 cusp tip	13	0.790	3.522	−0.026	2.126	0.482	0.996	1.636	0.491	1.770	0.458	0.019	0.664	1.044	1.921	0.082
#16 cusp tip	13	−0.161	1.603	− 0.257	1.731	0.885	1.843	1.759	0.509	1.482	0.047	0.397	0.702	1.494	1.385	0.018
#26 cusp tip	13	−0.083	1.571	− 0.190	1.716	0.87	1.940	2.018	0.756	2.386	0.185	0.224	0.867	1.051	2.002	0.185
ANS	13	−0.042	1.598	− 0.045	1.576	0.997	0.661	0.988	0.730	1.470	0.89	1.655	1.442	1.177	1.805	0.462
PNS	13	−0.178	1.080	0.092	0.882	0.492	3.263	1.736	1.100	2.428	0.015	1.723	1.487	1.618	1.508	0.859
A point	13	−0.027	1.518	−0.016	1.539	0.99	0.767	0.950	−0.107	2.054	0.18	1.409	1.250	1.447	1.093	0.94

*ANS* anterior nasal spine, *PNS* posterior nasal spine, *SD* standard deviation

**Table 3:** Differences in coordinate value and distance between Tv and T1 with PolyWorks Inspector™

		Coordinate value difference															Distance difference	
		Xv		X1			Yv		Y1			Zv		Z1			Tv and T1	
	n	Average	SD	Average	SD	*p*	Average	SD	Average	SD	*p*	Average	SD	Average	SD	*p*	Average	SD
Incisor root	13	−0.657	0.884	−0.551	0.915	0.765	−9.498	5.583	−9.597	5.651	0.964	58.596	8.086	58.848	7.659	0.936	0.820	0.694
#13 root	13	−17.639	1.749	−17.733	1.460	0.883	−7.312	6.825	−7.604	6.622	0.913	51.769	8.050	51.777	7.815	0.998	0.819	0.904
#23 root	13	16.208	1.621	16.401	1.454	0.752	−7.477	5.886	−7.805	5.734	0.887	51.706	8.526	51.660	8.078	0.989	0.817	1.196
#16 root	13	−29.700	2.270	−29.747	2.389	0.959	−9.119	7.143	−9.787	6.847	0.810	37.436	10.435	37.069	10.678	0.930	1.196	1.303
#26 root	13	28.993	2.451	28.921	2.730	0.944	−8.716	6.300	−9.482	6.117	0.756	35.991	9.303	36.135	9.043	0.968	1.022	1.661
ANS	13	−0.602	1.157	−0.440	0.989	0.705	0.091	6.152	−0.209	6.423	0.904	59.772	8.377	58.865	8.015	0.78	0.883	1.793
PNS	13	−1.104	2.046	−0.969	1.893	0.863	1.708	6.314	0.552	5.860	0.633	18.227	9.234	17.670	8.905	0.877	1.661	1.489
A point	13	−0.863	0.944	−0.804	0.828	0.866	−4.078	6.194	−4.453	6.243	0.879	57.691	8.034	57.723	7.693	0.992	0.860	1.071

*ANS* anterior nasal spine, *PNS* posterior nasal spine, *SD* standard deviation

**Table 4:** Distance difference between T1 and T2, T2 and T3, and T1 and T3

		T1 and T2		T2 and T3		T1 and T3		
	N	Average	SD	Average	SD	Average	SD	*p*
Incisor root	13	−0.109	1.037	−0.117	0.237	−0.529	0.982	0.352
# 13 root	13	0.393	0.930	−0.044	0.236	−0.165	0.617	0.089
#23 root	13	0.271	0.556	−0.057	0.197	−0.145	0.680	0.113
#16 root	13	−0.053	0.591	−0.016	0.183	−0.138	0.896	0.880
#26 root	13	0.354	1.066	0.010	0.322	−0.446	1.389	0.153
ANS	13	−0.823	1.811	−0.105	0.496	−0.821	1.449	0.316
PNS	13	0.083	0.770	−0.179	0.623	−0.043	0.845	0.741
A point	13	0.030	0.910	−0.005	0.164	−0.665	0.808	0.028

## Discussion

In this study, we report virtual surgical simulation with FaceGide® incorporating PCGs (including drilling holes for screws), PCMs and a customized final splint. In our series, the surgical transfer of the VSP by FaceGide® showed good accuracy, and the final position of the maxilla measured at the points associated with the root of the tooth (bone surface) was 0.94 ± 0.17 mm from the mean value. The ANS, PNS, and A point may show large differences between Tv and T1 because they are the sites of bone removal during the actual operation. However, the error was still approximately 1.01 ± 0.3 mm, even when these points were included.

With the introduction of CBCT, which reduces the hardware costs and radiation doses, 3D imaging can be used as a standard tool for diagnosis and treatment planning [16]. Although it is possible to obtain much information from this 3D diagnostic method, the IMS is generally used in conventional bimaxillary OGS. Increased use of the IMS can cause postoperative problems because the inherent thickness of the splint may result in a degree of autorotation after the splint’s removal [17]. Additionally, Perez et al. reported that the temporomandibular joint (TMJ) is not a discrete ball-and-socket joint. The mandibular condyle rotates and translates within the TMJ [18]. Therefore, repositioning the maxilla in relation to the position of the mandible may have several limitations. The mandible and maxilla can be fixed together with an IMS during OGS, but a certain amount of space can develop as a result of the mobility of the mandibular condyle. Therefore, the maxilla cannot be precisely positioned relative to the base of the skull using only an IMS, and the surgeon must take time to adjust it manually. In addition, inaccuracy of the IMS can arise from the model surgery stage. Model surgery depends on the accurate recording of the occlusion in the retruded position and the face-bow transfer to the articulator. These recordings both have inherent inaccuracy. Baily et al. measured the angulation of the occlusal plane to the Frankfort plane on a Hanau articulator and compared this with lateral cephalograms; they found a mean difference of 5 degrees, which corresponded to 70% of the error during the face-bow transfer [19]. Ellis et al. reported that the average case had an inaccuracy of almost 7 degrees in the angulation of the occlusal plane [20]. The accuracy of the 3D position of the upper first molar was highly variable using four different Hanau face-bows [21]. When using a conventional articulator for OGS, it is essential that the angle between the occlusal plane and the Frankfort horizontal plane for the patient is the same as the angle between the occlusal plane and the upper member of the articulator on the maxillary model. OGS using the FaceGide® system can reduce errors related to mock surgery because it does not use such an articulator during preoperative preparation.

The surgical method presented in this study using the FaceGide® system is not necessary for all patients with craniofacial deformity. Rustemeyer et al. reported that a 2D cephalometric analysis and a 3D mock operation are sufficient for accurate planning and will ensure good results for experienced surgeons [22]. We agree with this opinion, and if the patient has no facial asymmetry or requires only single-jaw surgery, conventional OGS can produce good results. However, if major 3D movements are indicated, including changes to the transverse occlusal plane or major rotation of the jaws, a navigation system should be chosen for complex 3D planning and controlling the position of the maxilla during surgery [23]. Most of the patients in this study had facial asymmetry. Therefore, the use of the FaceGide® system was recommended for surgery, and complex 3D movement of the jaws was performed. Our method of OGS using the FaceGide® system is very original, albeit not new. The principle of the FaceGide® system that we present is based on the combination of previously mentioned processes and is associated with predrilling determined by a reverse approach [24]. In a study by Xia et al., the final state was made into a medical replica after the VS, and the ready-made plates were bent according to the outline of this replica. During the actual operation, drilling for screw insertion was performed using the navigation system [24]. Use of the FaceGide® system is the same as the reverse approach of that reported by Xia et al., but customized miniplates and corresponding osteotomy guides (including drilling guides) are used. Similar processes involving predrilling and positioning osteotomy guides or prebent plates in OGS have been reported [25–28].

Ellis reported that the average accuracy of maxillary positioning in the horizontal plane deviated 2 mm from what was planned when external references were used, whereas the vertical accuracy ranged from 0.5 to 1 mm [29]. Jacobson et al. reported that a 2-mm or greater discrepancy was noted for 20 to 30% of 46 patients who underwent LeFort I osteotomy [30]. With the development of CAD and CAM, VSPs and 3D-printed navigation templates have been proposed as alternatives to conventional model surgery [31]. Sun et al. performed a clinical study using an orthognathic surgical template made from a 3D printer and VSP and reported that the mean vertical, lateral, and anteroposterior errors in the anterior maxillary region were 0.57 mm, 0.35 mm, and 0.5 mm, respectively [32]. Although our study shows a higher error than that of Sun et al.’s study, the difference may have been due to the use of different measurement methods, and our error was smaller than that reported in previous studies [29, 30] that used conventional methods. During the entire process, errors in surface rendering, data integration (merging dentition and CBCT data), and setting 3D coordinates in the virtual space or during guide, surgical splint and miniplate fabrication (3D printing or milling process) are related to accuracy. Accuracy can be improved with the use of a systemic process during surgical planning and preparation. Zinser et al. reported that the mean vertical, lateral, and anteroposterior errors compared with the anterior maxillary region were 0.23 mm, 0.04 mm, and 0.09 mm, respectively, and that the vertical, lateral, and anteroposterior errors compared with the posterior maxillary region were 0.15 mm, 0.04 mm, and 0.1 mm, respectively [33]. Our results are not comparable because we did not use the same measurement approach that was used by Zinser et al. However, in our study, the differences between the VS and the actual surgery were 0.26 mm, 0.47 mm, and 1.11 mm in the anterior maxillary region (incisor tip, #13 cusp tip and #23 cusp tip) and 0.02 mm, 1.6 mm, and 0.6 mm in the posterior maxillary region (#16 cusp tip, #26 cusp tip and PNS).

We are aware that this study may have limitations. The small number of patients in this retrospective study limits the ability to draw definite conclusions. One reason for the small sample size was the utilization of strict inclusion and exclusion criteria, which resulted in the exclusion of the majority of patients who underwent OGS in the department during the study period. However, image analysis using 3D comparison programs is highly reproducible and can yield significant results even with a small number of cases. There were some trial and error in the operation using the FaceGide® system. There were no significant differences between ΔTv and ΔT0, but in some cases, the Y coordinate value of the PNS was somewhat different from that of the other sites. Therefore, even when the operation is performed using this system, more attention should be paid when the posterior part of the maxilla is moved. In three patients, the maxilla was unstable after fixation, so the ready-made miniplates were added for reinforcement. In some cases in which the surgeon was unfamiliar with the newly developed system, wide-diameter screws were used because of a widening of the holes after drilling. However, this problem could be solved by drilling with a small-diameter round burr and self-drilling screws. PCGs with an arm that originates from the cusp of the teeth can also confirm the accuracy of the bone contact (Fig. 2). Minor mispositioning of the PCGs is impossible to detect by the naked eye and can result in erroneous cuts. Therefore, this type of PCG is believed to be more accurate than a bone-only supported guide because it is supported by both the bone surface and the cusp of the teeth, but further research regarding its accuracy is needed. The accuracy of OGS using CASS is influenced by reproduction of the VS in the actual surgery. Individual errors can originate from internal sources, including the CBCT image quality, file conversion process, computer design software, and interactions between mechanical components, and external sources, such as the adaption of the osteotomy guide, customized plate, and splints and the surgeon’s experience. The accumulation of such errors produces the total deviation between the planned and postoperative outcomes. However, this study demonstrates the suitable accuracy and stability of OGS using the FaceGide® system.

The facial surgery protocol using FaceGide® has advantages similar to those of other CASS systems. Digital diagnosis and VS data files can be transmitted to the surgeon and orthodontists for final adjustments [33]. Such exchanges have the advantage of allowing the creation of an interdisciplinary platform that centralizes the technical and surgical domains of expertise and produces financial and operative efficiency, all within a digital environment [31]. Because the site of the bone screw insertion is designed to avoid the root of the tooth, it is unlikely that root damage will occur when conventional methods are used. Some clinicians may claim that using CASS can expose the patient to increased radiation [34]. However, it can eliminate the need for additional radiographic examinations, which is indicated when there are doubts about the final surgical position. It is well known that the radiation dose of most CBCT systems used to acquire DICOM data is considered minimal [35]. There have been reports of the use of one piece of customized plate in the maxilla that have claimed accuracy [36–38]. However, the amount of titanium used in one-piece plates is greater than the amount of titanium used in conventional maxillary fixation methods. Hosoki et al. reported that the detailed mechanism of action of allergy and hypersensitivity to metallic materials is not known but is related to the total exposure to specific metallic ions [39]. In this study, four customized L-shaped miniplates were used, and they remained stable for more than one year without any bone changes. An excessive amount of titanium can cause a foreign body reaction. The volume of titanium used in our study is equal to or less than the volume of titanium used in the conventional fixation method of LeFort I osteotomy. Although not reported in this study, the PCMs were removed in three patients one year after surgery and did not cause clinical problems. Future studies involving PCM removal will be conducted. Unlike conventional surgery, the use of PCGs in our study allowed us to remove the necessary amount of bone, thereby increasing contact between bone segments. Our method was less invasive than conventional methods, and the patients recovered rapidly and were able to return quickly to normal life.

## Conclusions

The maxillary bone should be positioned according to a planned position during OGS to achieve a successful operation. We performed OGS using the FaceGide® system, which is a newly developed CASS system. The repositioning of the maxilla was clinically accurate, and stable results were maintained one year after the operation. Currently, the quality of the surgical result still depends on the skill of the individual surgeon in carrying out the surgical plan. However, surgeons with average experience will be able to achieve acceptable treatment results using the FaceGide® system (via a VSP and manufacturing of the related materials). In other words, 3D evaluation, virtual simulation, and CAD-CAM technology can benefit both doctors and patients. The development of digital technologies will continue to support the adoption of computer-assisted techniques in medicine and dentistry.

## Abbreviations

3D: Three-dimensional
CAD: Computer-aided design
CAM: Computer-aided manufacturing
CASS: Computer-aided surgical simulation
CBCT: Cone beam computed tomography
CNC: Computer numerical control
CR: Centric relation
IMS: Intermediate splint
OGS: Orthognathic surgery
PCG: Patient-customized osteotomy guide
PCM: Patient-customized miniplate
SD: Standard deviation
STL: Surface tessellation language
VSP: Virtual surgical plan
VTO: Visual treatment objective
